# Low‐carbohydrate, healthy‐fat eating: A cost comparison with national dietary guidelines

**DOI:** 10.1111/1747-0080.12534

**Published:** 2019-04-24

**Authors:** Caryn Zinn, Sylvia North, Katie Donovan, Chloe Muir, George Henderson

**Affiliations:** ^1^ School of Sport and Recreation, Human Potential Centre, Faculty of Health and Environmental Sciences Auckland University of Technology Auckland New Zealand

**Keywords:** cost, LCHF, low‐carbohydrate, healthy‐fat, national nutrition guidelines

## Abstract

**Aim:**

A low‐carbohydrate, healthy‐fat (LCHF) dietary approach has been demonstrated as an effective strategy for improving metabolic health; however, it is often criticised for being more expensive than following a dietary approach guided by the national, Ministry of Health nutrition guidelines. This study compared the cost of these two nutritionally replete dietary approaches for one day for a family of four.

**Methods:**

In this descriptive case study, one‐day meal plans were designed for a hypothetical family of four representing the average New Zealand (NZ) male and female weight‐stable adult and two adolescent children. National documented heights, a healthy body mass index range (18.5–25.0 kg/m^2^), and a 1.7‐activity factor was used to estimate total energy requirements using the Schofield equation. Total daily costs were compared based on food prices from a popular Auckland supermarket. Meal plans were analysed for their nutritional adequacy using FoodWorks 8 dietary analysis software against national Australian and NZ nutrient reference value thresholds.

**Results:**

The total daily costs were $43.42 (national guidelines) and $51.67 (LCHF) representing an $8.25 difference, or $2.06 per person, with the LCHF meal plan being the costlier option.

**Conclusions:**

We consider this increased cost for an LCHF approach to be negligible. In practice, less costly food items with similar nutrition qualities can be substituted to reduce costs further should this be a goal. The LCHF approach should therefore not be disregarded as a viable dietary approach for improving health outcomes, based on its perceived expense.

## Introduction

When it comes to the promotion of optimal health, prevention and management of chronic disease, we base our dietary guidance on the national Ministry of Health (MOH) food and nutrition guidelines.^1^ More recently, an alternate option for dietary guidance has emerged, that is a wholefood‐based approach, characterised by a reduced carbohydrate, higher natural fat intake, also termed low‐carbohydrate, healthy‐fat (LCHF). This approach is becoming increasingly employed in clinical practice as an equally suitable, and in some cases more effective management strategy for a variety of chronic conditions, in particular, diabetes.^2^, ^3^, ^4^ It has also been shown to be effective both in the short and long term for its beneficial outcomes on metabolic health.^5^, ^6^, ^7^, ^8^, ^9^ Eating according to the LCHF approach is often criticised as being more expensive than eating following standard dietary guidelines, yet there is no literature to draw on to confirm or refute this.

The cost of food plays a central role in determining food choices for New Zealanders. In a national 2010 survey, cost was highlighted as the main influencer of food and drink purchases by 75% of the population surveyed (n = 1740), regardless of ethnicity and neighbourhood deprivation.^10^ While the bulk of the literature alludes to a greater cost of healthy foods in general, compared with unhealthy foods, this might depend on the food classification systems used in studies. Cross‐sectional observational studies in Australia and Europe have shown foods considered to be healthier, defined by a higher nutrient density, lower energy density, or through meeting government guidelines, tend to cost more.^11^, ^12^, ^13^, ^14^ A systematic review and meta‐analysis of 10 countries, including New Zealand (NZ), found healthier diets, on average tend to cost US$1.48 more per day than unhealthy diets.^15^ In contrast, an NZ study investigating 10‐year trends in food costs defined healthy food by the degree of food processing, and found processed foods to be NZ$0.51 more expensive than minimally processed foods; however, the price difference between ultra‐processed and minimally processed foods was negligible.^16^

When it comes to comparing healthy eating approaches with each other rather than isolated foods or ultra‐processed food‐based diets, few studies have examined such cost variances. Wilson *et al.* 17 compared the cost differences between a nutritionally adequate, typical NZ male diet (excluding alcohol), and an Asian and a Mediterranean‐style dietary approach and found the latter two to be significantly less expensive than the former. A further cost comparison between Mediterranean, Paleo and Intermittent Fasting (IF) diets found no significant differences in costs between diets.^18^

Ultimately, where eating well is a key factor in promoting good health and the reduced risk of lifestyle‐related disease, a healthy diet not only needs to be nutritionally replete, and sustainable to follow long term, but also financially viable. There is no documented literature that we are aware of on the costs associated with the LCHF approach. With the growing widespread use and interest in this dietary approach, we decided that this was an important aspect to explore. The aim of this study was to compare the costs of a nutritionally replete one‐day meal plan for a family of four for two dietary scenarios: LCHF and MOH national nutrition guidelines.

## Methods

This descriptive study was conducted using a hypothetical case study scenario defined to represent a family of four living in Auckland, NZ. The family selected was designed to approximate the average Auckland household size according to the 2013 census data.^19^ The family included two weight‐stable adults (male and female) and two adolescent children (male and female) with no medically diagnosed health conditions. Participant characteristics were defined using anthropometric parameters to represent a healthy body mass index (BMI) (18.5–25.0 kg/m^2^)^1^ based on the estimated mean height of New Zealanders from each age and gender demographic.^20^ Individual nutrition requirements of the participants were determined using the Schofield equation for predicting basal metabolic rate^21^ and adjusted for gender, anthropometric information and a light‐to‐moderately active physical activity level (PAL, 1.7).^22^ Table 1 presents the demographic data used for the case studies.

**Table 1:** Family demographics

Family member	Age (years)	Height (cm)	Weight (kg)	BMI (kg/m^2^)	PAL	EER (kJ)	EER (Cal)
Adult male	45	176.0	72	23	1.7	12 085	2888
Adult female	45	162.9	60	23	1.7	9483	2266
Adolescent male	14	169.8	65	23	1.7	12 964	3098
Adolescent female	12	156.1	55	23	1.7	10 268	2454

BMI, body mass index; EER, estimated energy requirement; PAL, physical activity level.

Dietary comparison for the two approaches was conducted using an energy‐matched estimated one‐day's intake for each participant. Meal plans were analysed using the nutrient analysis programme FoodWorks 8 Professional Edition (Xyris Software, Australia). Meal plans representing the MOH nutrition guidelines were developed to be consistent with food group and serving recommendations for NZ adults and adolescents according to the Eating and Activity Guidelines for NZ Adults^1^ and Food and Nutrition Guidelines for Healthy Children and Young People.^23^ Macronutrient intake was aligned with the acceptable macronutrient distribution ranges (AMDR) to reduce risk of chronic disease (45–65% energy from carbohydrate, 15–25% energy from protein and 20–35% energy from fat with less than 10% energy from saturated fat).^22^ For the LCHF meal plans, macronutrient intake was established to align with the AMDR for protein (15–25% of energy). Carbohydrate intake was selected to provide 10–20% energy (60–120 g), with the remaining energy derived from wholefood sourced fat (55–70% of energy). There was no defined restriction for saturated fat intake. All micronutrients for the two diets were assessed on FoodWorks against national Australian and NZ nutrient reference value (NRV) thresholds.^22^

Types of food consumed were standardised across both diets to include traditional meals, to exemplify common dietary choices and to match local food availability of the wider NZ population. Food cost data were collected from shelf prices examined from a reputably inexpensive local supermarket, Pak 'n Save in Albany, Auckland, NZ, in October 2016.^24^ Food items selected for inclusion were based on the following criteria: (i) foods that we considered to be, generally, popular and acceptable, rather than any specialty or unusual food that would demand an acquired taste; (ii) lowest cost brands within a food category, apart from eggs, where free range was selected over conventional eggs, for ethical reasons; and (iii) fresh vegetables were chosen in preference over frozen except for green beans, brussel sprouts and spinach. Costs for each one‐day sample meal plan for all participants were calculated by price per weight of food product consumed. Where price data were acquired for uncooked weight of foods (such as meat and rice), the weight of foods was adjusted for the cooked version.

## Results

Tables 2 and 3 present the sample meal plans of the male and female case studies for both dietary approaches, and their macronutrient and energy composition, respectively.

**Table 2:** MOH nutrition guidelines and LCHF meal plans for each family member

	MOH nutrition guidelines meal plans			LCHF meal plans			
Adult male	Adolescent male	Adult female	Adolescent female	Adult male	Adolescent male	Adult female	Adolescent female
Breakfast				Breakfast			
*Toast with tuna/avocado*: Mixed grain bread, three slices; margarine, 2 tsp; tuna, 90 g; Mixed grain bread, three slices; avocado, ½; trim milk, 1 c	*Fruity oats*: Oats, raw, 1 ¾ c; trim milk, 1 c; banana, 1 medium; frozen blueberries, ½ c	*Fruity oats*: Oats, raw, ⅔ c; trim milk, 150 mL; banana, ½ medium; sunflower seeds, 15 g; iodised salt, ¼ tsp	*Fruity oats and smoothie*: Oats, rolled, raw, ½ c; reduced fat fruit yoghurt, ½ c; frozen blueberries, ½ c; *Smoothie*: Trim milk, 1 c; reduced fat fruit yoghurt, ½ c; banana, 1 medium; frozen blueberries, oats, rolled, raw, ¼ c	*Scrambled eggs with vegetables*: Egg, 3 size 6; mushroom, 15 g; tomato, 100 g; onion, ¼ c; butter, 1.5 tbsp; iodised salt, ⅛ tsp	*Scrambled eggs with vegetables*: Egg, 4 size 6; mushroom, 17 g; tomato, 120 g; onion, ¼ c; butter, 1.5 tbsp; iodised salt, ⅛ tsp	*Scrambled eggs with vegetables*: Eggs, 3 size 6; mushroom, 15 g; tomato, 75 g; onion, ¼ c; butter, 3 tsp; cheese, cheddar, 40 g; iodised salt, ⅛ tsp	*Fruit smoothie*: Whole milk, 1 c; yoghurt, full fat Greek‐style, 1 c; frozen blueberries, ½ c; banana, ½ medium; sunflower seeds, 2 tbsp
Lunch				Lunch			
*Two Ham and salad sandwiches:* Mixed grain bread, four slices; ham, 95 g; lettuce, ½ c; tomato, 30 g; carrot, ⅓ c; mayonnaise, reduced fat, 3 tsp; Muesli bar, multigrain; Apple, 1 fruit	*Two Ham and salad sandwiches:* mixed grain bread, four toast slices; margarine, 3 tsp; spinach, raw, ½ c; cheese, Edam, 30 g; ham, 95 g; Muesli bar, multigrain; Banana, 1 medium	*Beef and rice salad:* Mesclun salad, 1 c; brown rice, 1.5 c; lean beef, 80 g; reduced fat vinaigrette dressing, 1 tbsp; Multigrain crackers, 6 biscuits; cheese, Edam, 40 g	*Beef and cheese pitas:* Wholemeal pita, 2; lean beef, 60 g; mesclun salad, ½ c; carrot, raw, ½ c; mayonnaise, reduced fat, 3 tsp Banana, 1 medium Muesli bar, multigrain, 50 g	*Chicken and vegetable salad*: Chicken thigh, roasted, 130 g; sweet potato, 110 g roasted in 1 ½ tbsp olive oil; ⅛ tsp iodised salt; pumpkin seeds, 2 tbsp; sunflower seeds, 1 tbsp; spinach leaves, 1 c; capsicum, 83 g; cheese, cheddar, 80 g	*Chicken and vegetable salad:* Chicken thigh, roasted, 100 g; sweet potato, 145 g roasted in 2 tbsp olive oil; ⅛ tsp iodised salt; capsicum, 166 g; carrot, 110 g; avocado, ½; cheese, cheddar, 50 g	*Roast beef salad*: Beef brisket, braised, 80 g; mesclun salad, 1 c; tomato, 120 g; cucumber, ½ c; carrot, ½ c; olive oil, 1 tbsp; pumpkin seeds, 2 tbsp	*Roast beef salad*: Beef brisket, braised, 80 g; mesclun salad, 1 c; tomato, 120 g; cucumber, 1 c; carrot, 1 c; olive oil, 1.5 tbsp; pumpkin seeds, 2 tbsp; cheese, cheddar, 50 g
Dinner				Dinner			
*Roast chicken and veg*: Chicken breast, 130 g; broccoli, 1 c cooked; potato, 2 c cooked; spinach, 1 c raw; olive oil, 1 tbsp	*Roast chicken and veg*: Chicken breast, 130 g; potato, 2 c cooked; broccoli, 1 c cooked; pumpkin, 1 c cooked; spinach 1 cup, raw; olive oil, 1 tbsp	*Roast chicken dinner*: Chicken breast, 80 g; pumpkin baked, ⅔ c; spinach steamed, ½ c; broccoli cooked, 1½ c; potato, 1 c; olive oil, 3 tsp; iodised salt, ¼ tsp	*Roast chicken dinner*: Chicken breast, 90 g; potato, cooked 1 c; broccoli, cooked, 1 c; pumpkin, baked, ½ c; spinach, steamed, ½ c; olive oil, 2 tsp; iodised salt, ¼ tsp	*Roast chicken and veg*: Chicken thigh, 130 g; pumpkin, 1 c cooked; broccoli, 1 c cooked; spinach, steamed, ½ c; olive oil, 2 tbsp	*Roast chicken and veg*: Chicken thigh, 130 g; pumpkin, 1 c cooked; broccoli, 1 c cooked; spinach, steamed, ½ c; olive oil, 2 tbsp	*Roast chicken dinner*: Chicken leg, 80 g; broccoli, cooked, 1 ½ c; pumpkin, baked, 1 c; spinach, steamed, ½ c; olive oil, 3 tsp; iodised salt, ¼ tsp	*Roast Chicken dinner*: Chicken leg, 90 g; broccoli, 1 c cooked; pumpkin, baked, 1 c; spinach, steamed, ½ c; olive oil, 2 tsp.; iodised salt, ¼ tsp
Snacks				Snacks			
Yoghurt, plain, low fat, ½ cup; banana, 1 medium chopped; Multigrain crackers, 10 biscuits; cream cheese, reduced fat, 80 g Kiwifruit, 1 fruit	Smoothie: trim milk, 250 mL; banana, 1 medium Mixed grain bread, two toast slices; jam, 2 tsp (on one slice); natural peanut butter, 2 tsp (on one slice) Fruit yoghurt, ½ c; almonds, 7 g; raisins, 13 g	Smoothie: trim milk, 200 mL; kiwifruit, 2 fruits; oats, raw ⅓ c Yoghurt, plain, low fat, 1 c; apple, 1 fruit, chopped	Yoghurt, fruit, low fat, ½ c; apple, 1 chopped; almonds, 12 g Mixed grain bread, two slices; margarine, 2 tsp; marmite spread, 1 tsp; cheese, Edam 40 g	Apple, 2 fruits, chopped, dipped in natural peanut butter, 3 tbsp Yoghurt, Greek‐style, full fat, 1 c; frozen blueberries, 1 c Brazil nuts, 38 g	Yoghurt, Greek‐style, full‐fat, 1 c; frozen raspberries, ½ c; natural peanut butter, 3 tbsp Almonds, 24 g; sunflower seeds, 30 g Banana, 1 fruit	Smoothie: whole milk, 250 mL; kiwifruit, 1 fruit Yoghurt, full fat Greek‐style, 1 c; sunflower seeds, 30 g; almonds, 18 g	Cucumber, Lebanese, 1, with spread natural peanut butter, salted, 3 tsp Almonds, 18 g; cashew nuts, 50 g Apple, 1 fruit

LCHF, low‐carbohydrate, healthy‐fat; MOH, Ministry of Health.

**Table 3:** Total energy and macronutrient distribution for the MOH nutrition guidelines, and LCHF meal plans

	MOH nutrition guidelines				LCHF			
Nutrient breakdown	Adult male	Adult female	Adolescent male	Adolescent female	Adult male	Adult female	Adolescent male	Adolescent female
Energy								
kJ	12 742.2	9494.0	13 035.2	10 679.9	12 773.1	9442.2	13 045.2	10 310.3
Cal	3045.5	2269.1	3115.5	2552.6	3052.8	2256.7	3117.9	2464.1
Carbohydrate								
g	352.9	277.5	394.2	325.1	102.3	63.1	103.9	114.3
%	46.2	48.8	50.3	50.6	13.0	10.8	12.9	18.0
Protein								
g	158.6	129.3	163.0	139.6	150.6	131.5	142.6	122.8
%	21.2	23.2	21.3	22.2	20.0	23.7	18.6	20.3
Fat								
g	96.1	60.1	82.1	59.6	220.5	159.3	230.7	161.8
%	27.9	23.4	23.3	21.7	63.9	62.4	65.4	58.1
Saturated Fat								
g	23.9	18.9	21.3	18.7	70.1	62.2	62.4	56.2
%	6.9	7.4	6.0	6.3	20.3	24.0	17.7	20.2

LCHF, low‐carbohydrate, healthy‐fat; MOH, Ministry of Health.

**Figure 1:**
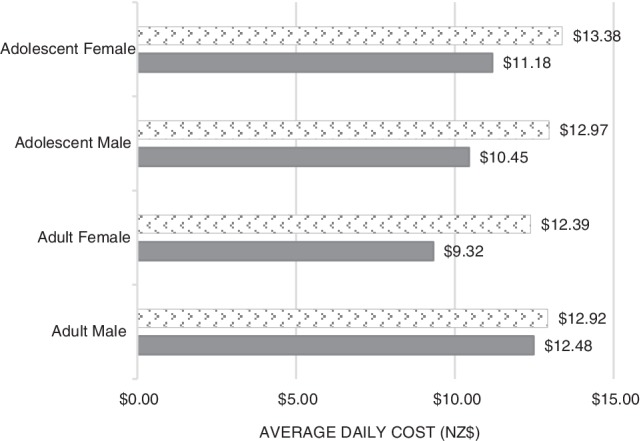
Cost comparison analyses for one‐day sample meal plans (LCHF, low‐carbohydrate, healthy‐fat; MOH, Ministry of Health; NZ, New Zealand). (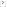) LCHF; (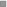) MOH national guidelines.

Cost comparisons for the one‐day meal plans are presented in Figure 1. The total combined costs for the diets of all four family members were $43.42 for the MOH nutrition guidelines (average cost per person: $10.86) and $51.67 for LCHF (average cost per person: $12.92). The difference between the two dietary approaches was $8.25, with the LCHF diet being the more expensive option. Taken as an average cost per family member, this amounted to $2.06.

All versions of the meal plans were replete for all the micronutrients as compared against their recommended dietary intake (RDI) or specific dietary target (SDT) thresholds apart from the mineral selenium, which reached 95% of the RDI for the adolescent male in the MOH guidelines plan (see Appendix I). The maximum SDT for sodium for the adult male and female, and the adolescent female for the MOH guidelines meal plans was exceeded.

## Discussion

This is the first study to compare the food costs of a nutritionally replete meal plan for a wholefood‐based LCHF approach with that of an MOH national nutrition guidelines‐based approach. The key finding was that in this instance, the LCHF was the costlier set of meal plans of the two amounting to an additional $8.25 per day for a family of four, or an average of $2.06 per person. Extrapolated to one week totals $361.69 and $303.94 for the LCHF and MOH plans, respectively; however, these figures should be interpreted with caution. Purchased meals, snacks and beverages consumed outside of the home environment, as well as different meal varieties could either overestimate or underestimate costs over the week for both dietary conditions.

Our findings differed from those reported by Wilson *et al.* 17 This research group reported an Asian and Mediterranean meal plan at NZ$4.95 per day and NZ$5.64 per day, respectively; with both meal plans less costly than that of a typical NZ male (as guided by national survey data from the National Nutrition Adult Nutrition Survey),^25^ costed at $17.29 per day. In this NZ study, nutrient thresholds were assessed against estimated average requirements (EARs), that is the intake required to meet the needs of half of the population; hence it represented a 1000kJ lower dietary energy requirement than that used in our study. Researchers also used a selection of micronutrients for comparison rather than the full spectrum as we did (i.e. vitamin B1—thiamine—to represent all the B vitamins, upper limits for sodium and vitamin A) and undertook analyses for males only.

In contrast, our cost findings for LCHF ($12.92) were similar to those reported by Park who conducted a dietary adherence study using weighed dietary records with actual participants. Park reported no significant difference between average daily costs of three diets: Mediterranean diet: $11.27; Paleo diet: $12.85 and IF on a non‐fasting day, where the participants could eat whatever they wished: $12.06. One would assume a cost similarity between Paleo and LCHF dietary approaches as they share a common dietary philosophy, that is a focus on the consumption of whole unprocessed foods. However, this argument does not hold true considering the similar costs seen for IF on a non‐fasting day.^17^, ^18^

Despite the costlier outcome for LCHF meal plans in this study, there are several important points that warrant consideration when it comes to estimating the cost of different diets, especially LCHF. The first of these is that whichever style of eating you adopt, there are mechanisms for reducing the cost of foods, even when foods are matched for nutrient composition. For example, extra virgin olive oil, fresh salmon, macadamia nuts and broccolini, foods often used with LCHF but not limited to it, would be considered expensive options, yet their less costly, approximate nutrient‐equivalents such as standard olive oil, sardines, linseed and frozen spinach are substantially cheaper. Ethical perspectives could also alter food costs. In this exercise, we used free‐range eggs (eggs were only used in the LCHF plan); however, using standard caged‐eggs would have been a less costly option, bringing the daily cost of the one‐day LCHF plan down by $2.00, and thereby, reducing the daily difference between the plans from $8.25 to $6.25.

In the context of the LCHF approach, there are some foods known to be less expensive than their equivalent following an MOH nutrition guidelines approach. For example, standard mince, rump steak and chicken thighs or wings with skin all have a higher natural fat content and are less costly than leaner cuts of meat such as premium mince, eye fillet steak and skinless chicken breasts. This point was reflected in our work as the ‘Meats’ food group comparative costs for the family were $16.41 for the MOH approach, and $12.67 for the LCHF approach. On the other hand, there are some foods known to be more expensive compared with their non‐LCHF counterparts, the notable one being almond flour (almond meal) *versus* standard white flour, an ingredient frequently used as a lower carbohydrate alternative for baking. In this case, there is also a vast difference between the nutritional content of these two flours, with almond flour being nutritionally superior.^26^ In these meal plans, we did not include any homemade baking products, and have therefore not captured this as a significant cost difference.

When other food groups were compared, there were similarities noted in the total amounts as follows: ‘fruit’: $5.25 *versus* $5.04; ‘dairy’: $5.27 *versus* $6.35; ‘fats, oils, spreads’ $1.34 *versus* $2.26; for the MOH plans and the LCHF plans, respectively. The main cost discrepancies were noted in the remaining food groups, ‘nuts and seeds’, ‘grains’ and ‘vegetables’, and were largely due to the different proportions of these foods used in the two sets of meal plans. The cost differences were as follows: ‘nuts and seeds’: 87c *versus* $8.64; ‘grains’: $6.49 *versus* $0 and ‘vegetables’: $7.80 *versus* $16.58, for the MOH *versus* LCHF plans, respectively. The total cost difference between these food groupings was $10.06, with LCHF being costlier than MOH. The greater use of nuts, seeds and vegetables in the LCHF plans was a necessary trade‐off from the absence of carbohydrate‐heavy grains, to ensure the macronutrient, micronutrient and fibre thresholds were met. The grains selected for the MOH plans were all wholegrain, rather than their known less costly refined counterparts. Despite this, grain‐based foods still tend to be a less costly option than nuts, seeds and vegetables based on the proportions used to satisfy certain nutrient requirements, in particular fibre and certain B vitamins. Modelled as family eating behaviours, our analysis included family‐style meals that allowed for strategic shopping, meal planning, purchasing in bulk and avoiding waste; known strategies to make weekly shopping affordable.^27^ In our study, foods were selected for both nutrition approaches equally with general affordability in mind. We selected low cost brands, seasonal fresh produce and frozen alternatives to more expensive fresh varieties. Whether either style of eating is considered affordable for low‐income families is important, but beyond the scope of this work.

The second important point to consider is the value people place on the foods they purchase in relation to their overall health. While LCHF may be a costlier dietary approach in this instance, for some the additional $2.06 per person per day (which accumulates, hypothetically, to $57.69 per week for the whole family) may be viewed as a worthy investment for the perceived health benefits and overall reduction in health‐related costs it may imply. Food purchasing is a complex issue; one only needs to consider the extent of discretionary food and beverage spending (i.e. weekly coffee consumption in 2015 ranged from $12.09 to $14.02 per person in different regions of NZ^28^) to realise that food purchasing behaviours can be influenced by both affordability (or lack of) and priorities, which differs markedly between individuals and families.

We were able to achieve nutrient‐replete diet plans for both dietary approaches (apart from the mineral selenium for the adolescent boy in the MOH guidelines plan—which can be remedied by the addition of one Brazil nut). This finding corroborates with previous LCHF plan nutrient analyses.^29^ MOH national nutrition guidelines promote a carbohydrate‐dominant, lower fat dietary approach, with adults and adolescents advised to eat a minimum of six servings of grain‐based foods daily as a main source of dietary energy (in the form of carbohydrate) as well as fibre, vitamins (B group vitamins excluding B12 and E) and minerals (magnesium, calcium, iron, zinc and selenium).^1^ Alternate low carbohydrate choices are believed to raise the cost of obtaining these essential nutrients either resulting in nutritional inadequacy or additional food cost.^30^ This was shown to some extent in our work, despite previous nutrient‐driven cost analyses showing LCHF‐appropriate wholefoods such as eggs, meat, dairy, dry beans, nuts, seeds, vegetables and fruit, among the lowest cost food sources of protein, fibre, vitamin A, vitamin C, calcium, iron and potassium.^31^

Our study had several limitations; however, none of them single out or bias any one style of eating but rather apply to both LCHF and MOH nutrition guidelines equally. The first is that only a one‐day sample meal plan was examined and therefore does not consider the variety that would usually be seen over the course of a week. As a result, the application of these findings to larger and more diverse groups over a longer duration is limited.

Another limitation was that food costs were determined from off‐shelf price information from an Auckland supermarket over a one‐week period. This did not account for usual budgeting strategies that involve taking advantage of food specials one might see at a different supermarket. Neither did it include the purchase of foods from vegetable shops, markets and the butcher, where often foods can be obtained more inexpensively, or foods from vegetable or herb gardens. However, this is not necessarily problematic, in that the aim of this study was not to undertake an exercise in cost‐effectiveness or affordability of food in general, but rather to compare the costs of two eating approaches. These limitations can be applied equally to both nutrition approaches and does not bias seasonal price or food choice variation.

The fact that these were theoretical case studies was a limitation as food preference was unable to be considered in food selection. Furthermore, there was potential for researcher bias in determining menu items and ‘typical’ food choices for the theorised participants.

A strength of the study is that an accurate, professional and local food composition database for dietary analysis was used. A further strength was the exclusion of specialty, fortified and unpopular foods in food selection to avoid bias towards nutrient density in any one dietary approach.

In conclusion, this study demonstrated that a nutrient‐replete LCHF meal plan was costlier in this instance than an MOH nutrition guidelines plan for a family of four. Whether $2.06 per person per day is considered a minor or a major difference in costs is subjective. Either way, we do not believe this constitutes a meaningful enough difference to warrant disregarding LCHF as a viable dietary approach for improving health outcomes, based on its perceived expense.

## Funding source

CM, one of the study authors received funding to assist with the study data collection and analysis from a contestable Graduate Assistantship grant supplied by AUT.

## Conflict of interest

Dr CZ has co‐authored four books on the topic of the LCHF dietary approach. All other authors declare no conflict of interest.

## Authorship

CZ and GH contributed to the study idea and design. CM undertook the data collection. CM, SN, KD and CZ contributed to the data analysis and interpretation. All authors contributed to the development of the manuscript. All authors approved the final manuscript and declare that the content has not been published elsewhere.

## Appendix I 1

**Table A1:** Micronutrient analysis for MOH nutrition guideline and LCHF diet plans for each family member

	MOH nutrition guidelines								LCHF							
Micronutrients	Adult male	% RDI (unless otherwise specified)	Adult female	% RDI (unless otherwise specified)	Adolescent male	% RDI (unless otherwise specified)	Adolescent female	% RDI (unless otherwise specified)	Adult male	% RDI (unless otherwise specified)	Adult female	% RDI (unless otherwise specified)	Adolescent male	% RDI (unless otherwise specified)	Adolescent female	% RDI (unless otherwise specified)
Thiamin (mg)	3.3	274	2.4	220	2.5	207	2.7	305	1.4	114	1.4	131	1.5	129	1.4	160
Riboflavin (mg)	5.2	402	3.3	296	3.7	282	3.9	434	3.4	262	3.7	338	3.7	286	3.1	355
Niacin (mg)	48.1	NA	21.1	NA	25.9	NA	22.0	NA	23.2	NA	10.4	NA	29.7	NA	13.1	NA
Niacin equivalents (mg)	81.3	508	48.0	343	59.0	369	46.6	389	59.5	372	40.8	291	65	406	40	333
Vitamin C (mg)	218.6	486	443.6	986	191.8	480	165.3	413	307.7	684	278.7	619	438.8	1097	185	462
Vitamin B6 (mg)	7.3	563	3.6	280	4.3	333	3.6	360	3.0	232	2.24	172	4.6	354	2.4	237
Vitamin B12 (μg)	8.5	354	5.9	246	6.2	257	5.4	299	7.7	320	6.8	285	7.3	302	4.7	259
Folate (dietary folate equivalents, μg)	1373.9	342	518.1	130	473.5	118	796.6	266	737.3	184	6.37	159	692.4	173	467.2	156
Total vitamin A equivalents (μg)	963.2	107	1760	252	1994.7	222	2048.7	341	3881.7	431	3803.4	543	4300.1	478	4225.2	704
Magnesium (mg)	552.2	131	593.9	186	766.6	187	592.4	247	630.7	150	563	176	541.1	132	664.3	277
Calcium (mg)	1236.4	124	1723.5	172	1913.5	147	1899.7	146	160.4	160	1630.8	163	1371.8	106	1672.1	129
Potassium (mg)	6399.7	136^a^	5389.3	115^a^	7254.0	154^a^	5426.0	115^a^	5199.8	111^a^	4494.4	96^a^	5880.7	125^a^	4930.2	105^a^
Phosphorus (mg)	2571.7	257	2507.2	251	2882.6	231	2414.5	193	2573.6	257	2375.4	238	2407.3	193	2326.7	186
Iron (mg)	25.6	319	18.4	102	26.1	237	19.0	237	18.8	235	18.7	104	16.3	149	17.7	221
Zinc (mg)	16.4	117	13.7	171	15.3	117	15.5	258	20.1	144	18.5	231	19.3	148	19.5	326
Selenium (μg)	125.2	179	60.5	101	66.8	95	78.7	157	572.5	818	95.1	159	108.6	155	62.9	126
Iodine (μg)	151.8	101	195.5	130	156.2	104	121.4	101	194.7	130	183.2	122	208.2	139	125.3	104
Sodium (mg)	3981.6	199^b^	2211.5	111^b^	1761.8	88^b^	2370.6	119^b^	1935.8	97^b^	1520.0	76^b^	1707.3	85^b^	1575.4	79^b^
Fibre (g)	61	161^a^	45	159^a^	66	173^a^	58	208^a^	39	103^a^	30	107^a^	39	103^a^	36	130^a^

a Potassium and fibre are presented as a percentage of the minimum SDT.

b Sodium is presented as a percentage of the maximum SDT.

NA, not applicable; LCHF, low‐carbohydrate, healthy‐fat; MOH, Ministry of Health, RDI, recommended dietary intake; SDT, suggested dietary target.
